# Nuclear exosome HMGB3 secreted by nasopharyngeal carcinoma cells promotes tumour metastasis by inducing angiogenesis

**DOI:** 10.1038/s41419-021-03845-y

**Published:** 2021-05-28

**Authors:** Kaiwen Zhang, Dong Liu, Jianmei Zhao, Si Shi, Xin He, Peng Da, Yiwen You, Bo You

**Affiliations:** 1 Department of Otolaryngology Head and Neck Surgery, Affiliated Hospital of Nantong University, Xisi Road 20, Nantong, 226001 Jiangsu Province China; 2 Institute of Otolaryngology Head and Neck Surgery, Affiliated Hospital of Nantong University, Xisi Road 20, Nantong, 226001 Jiangsu Province China; 3 School of Life Science, Key Laboratory of Neuroregeneration of Jiangsu and Ministry of Education, Co-innovation Center of Neuroregeneration, Nantong University, Nantong, 226001 China; 4 Molecular Detection Center, Affiliated Hospital of Nantong University, Xisi Road 20, Nantong, 226001 Jiangsu Province China; 5 Department of Pathology, Affiliated Hospital of Nantong University, Xisi Road 20, Nantong, 226001 Jiangsu Province China

**Keywords:** Metastasis, Tumour angiogenesis

## Abstract

Distant metastasis accompanied by angiogenesis is the main cause of nasopharyngeal carcinoma (NPC)-related death. Nuclear exosomes (nEXOs) are potential tumour biomarkers. High mobility group box 3 (HMGB3), a nuclear protein, is known to be overexpressed in cancers. However, its role in NPC has not been elucidated. Here, we explore for the first time the function of nEXO HMGB3 in tumour angiogenesis involved in NPC metastasis using a series of in vitro experiments with NPC cell lines and clinical specimens and in vivo experiments with tumour xenograft zebrafish angiogenesis model. We found a high expression of HMGB3 in NPC, accompanied by the formation of micronuclei, to be associated with metastasis. Furthermore, the NPC-secreted HMGB3 expression was associated with tumour angiogenesis. Moreover, HMGB3-containing nEXOs, derived from the micronuclei of NPC cells, were ingested by the human umbilical vein endothelial cells (HUVECs), and accelerated angiogenesis in vitro and in vivo. Importantly, western blotting and flow cytometry analysis showed that circulating nEXO HMGB3 positively correlated with NPC metastasis. In summary, nEXO HMGB3 can be a significant biomarker of NPC metastasis and provide a novel basis for anti-angiogenesis therapy in clinical metastasis.

## Introduction

Nasopharyngeal carcinoma (NPC) has a high incidence of head and neck squamous cell carcinoma (HNSCC) and is common in southern China^1^. Early detection and timely radiochemotherapy are essential for improving the survival rate of patients with NPC^2^. However, in many patients with advanced NPC, current radiochemotherapy has limited benefits. A large number of such patients are accompanied by distant metastasis and poor treatment outcomes^3,4^.

It is widely believed that tumour angiogenesis allows tumour cells to enter the blood circulation^5^. Angiogenesis involves angiogenic factors that stimulate endothelial cells, endothelial cell proliferation, germination and migration, lumen formation, vascular maturation and stabilisation and degrade vascular basement membranes^6^. The newly formed blood vessels provide a favourable environment for promoting tumour growth and metastasis^7,8^. Angiogenesis plays an important role in tumour progression and metastasis, and anti-angiogenesis is a clinically significant antitumor strategy^9^. Therefore, there is an urgent need to explore the molecular mechanisms of tumour angiogenesis.

Extracellular vesicles(EVs) are important biological messengers for intercellular communication, including specific subsets such as microvesicles, exosomes or apoptotic bodies^10,11^. Among them, exosomes are closely associated with tumour angiogenesis^12^. Exosomes are bilayer lipid structures ranging in size from 30 to 150 nm and contain proteins, lipids, and various types of nucleic acids^13^. Exosomes are released into the extracellular space, and the extracellular exosomes then bind to the receptor cell membrane, causing biological changes in the receptor cells^14^. Recently, accumulating evidence has revealed that exosomes secreted by tumour cells play a key role in tumour metastasis and drug resistance by accelerating angiogenesis^15,16^. Proteins contained in tumour-secreted exosomes shuttle from cell to cell to affect the genome-wide gene expression of the recipient cell.

Nuclear exosomes (nEXOs), abundant in nuclear proteins and genomic DNA, are a type of exosomes^17^. A theory suggests that nEXOs are derived from micronuclei^18^. Micronuclei, a hallmark of cancer, are associated with improper nuclear segregation^19^. HNSCC contains a broad range of somatic copy number alterations (SCNAs), which are related to altered segregation^20^. In summary, nEXOs can be characterised as important biomarkers of NPC.

Compared with healthy cells, cancer cells secrete a high amount of nEXOs^19^. High mobility group box 3 (HMGB3), belonging to the high mobility group (HMG) family, is a nuclear protein that is widely found in eukaryotes^21^. The HMG family consists of four members: HMGB1, HMGB2, HMGB3 and HMGB4^22^. HMGB3 is a multifunctional protein with different roles. It can bind to the chromatin in an atypical DNA structure (e.g., single-stranded DNA) and bend the DNA to increase its flexibility^23–25^. Unlike HMGB1 and HMGB2, HMGB3 is expressed at low levels in normal cells but often overexpressed (up to 20-fold) in cancer cells^26^. Moreover, HMGB3 is a marker of haematopoietic stem cells, and hence, HMGB3-deficient cells cannot regenerate bone marrow for a long time^27^. Notably, HMGB3 has been an attractive target for a wide variety of cancers, but HMGB3-targeted studies for NPC and exosomes containing HMGB3 have not been reported.

Herein, our clinical data suggested that HMGB3 expression was aberrant in NPC tissues and affected patient outcomes; notably, patients exhibited micronuclei formation and increased HMGB3 expression in the extracellular matrix. In tumour xenograft angiogenesis models and clinical samples, HMGB3 was closely correlated with the microvessel density (MVD), indicating that changes in HMGB3 levels in tumour cells regulate angiogenesis. Importantly, we identified that HMGB3-containing nEXOs derived from the micronuclei of NPC cells can be ingested by the vascular endothelial cells, thereby promoting metastasis by inducing angiogenesis. The findings of the present study indicate nEXO HMGB3 as a biomarker of NPC metastasis and therefore, may advance the development of novel anti-angiogenesis therapy for tumour metastasis.

## Materials and methods

### Cell lines and cell culture

Human umbilical vein endothelial cells (HUVECs), human NPC cell lines (CNE1, CNE2, 5–8 F and 6–10B) and the normal nasopharyngeal epithelial cell line NP69 were all cultured at the Institute of Otorhinolaryngology Head and Neck Surgery, Affiliated Hospital of Nantong University (Jiangsu, China). HUVECs and NPC cell lines were cultured in RPMI 1640(Biological Industries Israel Beit-Haemek, 01–100-1ACS) supplemented with 10% foetal bovine serum (FBS; Biological Industries Israel Beit-Haemek, 04–001-1ACS). NP69 cells were cultured in K-SFM (Thermo Fisher Scientific, 17005–042). All cell lines were identified by short tandem repeat (STR) fingerprinting, and the mycoplasma contamination was tested negatively before the experiment.

### Immunofluorescence

Cells were seeded in 15-mm glass bottom cell culture dish (NEST, 801002) at a density of 5 × 10^4^ cells. After a day, cells were washed thrice with phosphate-buffered saline (PBS; Gibco, 10010023) and fixed with 4% paraformaldehyde for 20 min. After removal of 4% paraformaldehyde, the cells were washed with PBS three times and incubated with 0.2% Triton X-100 (Beyotime, ST795) at room temperature for 10 min. After removal of 0.2% Triton X-100, the cells were washed thrice with PBS and incubated with the primary antibodies overnight at 4 °C. Primary antibodies used included anti-CD63 (cat:25682-1-AP, Proteintech, China). A day later, the cells were washed to remove the primary antibody and incubated with the fluorescently labelled secondary antibodies for 1 h in the dark. Anti-fluorescent attenuators containing Hoechst (Thermo Fisher Scientific, 62249) stain were then added to capture images.

### Western blot analysis

Cells and exosomes were lysed and collected in a protein lysate, and the concentration was measured using a BCA kit (Thermo Fisher Scientific, 23327). Protein samples (30 μg) were electrophoresed, transferred to a nylon membrane and blocked with a blocking buffer. Finally, the cells were incubated with the primary antibody at 4 °C overnight. The antibodies used were anti-HMGB3 (cat:D160490, Sangon Biotech, China), anti-flotillin-1 (cat:ab41927, Abcam, USA), anti-actinin-4 (cat:ab108198, Abcam, USA), anti-Alix (cat:ab186429, Abcam, USA), anti-CD9 (cat:ab92726, Abcam, USA), anti-GAPDH (cat:10494-1-AP, Proteintech, China), anti-E-cadherin (cat:A3044, ABclonal, China), anti-Vimentin (cat:10366-1-AP, Proteintech, China), anti-N-cadherin (cat:A3045, ABclonal, China), anti-HMGB1 (cat:10829-1-AP, Proteintech, China), anti-HMGB2 (cat:14597-1-AP, Proteintech, China) and anti-HMGB4(cat:12787-1-AP, Proteintech, China). Following incubation with a goat anti-rabbit secondary antibody, the immunoreactive proteins were detected with ECL western blotting detection reagents (Millipore, WBKLS0500).

### qRT-PCR

Total RNA was extracted using the TRIzol reagent (Thermo Fisher Scientific, 15596018) on ice, and samples were resuspended in RNAse-free water. Reverse transcription using a reverse transcriptase kit (Thermo Fisher Scientific, K1622) and qRT-PCR were conducted using the SYBR Green PCR Master Mix (Roche, 04913914001). The sequences of all the indicated primers are as listed below:

hmgb3-forward: TTTTCCAAGAAGTGCTCTGAGA

hmgb3-reverse: TTCTTCTTCTTGCCTCCCTTAG

### Transfection using plasmids and lentiviral vectors

Three short hairpin RNAs (shRNAs) against the human HMGB3 and their negative controls were constructed and generated by GeneChem (Shanghai, China). The sequences are as listed below.

shHMGB3-1: gatcccGTCCGGGAAAGAGAAATCTAActcgagTTAGATTTCTCTTTCCCGGACtttttggat

shHMGB3-2: gatcccAAGGAGAAGTATGAGAAGGATctcgagATCCTTCTCATACTTCTCCTTtttttggat

shHMGB3-3: gatcccAAGGAAAGTTTGATGGTGCAActcgagTTGCACCATCAAACTTTCCTTtttttggat

6–10B, NP69 and CNE2 cells were transfected with LV-HMGB3-GFP (GV218; GeneChem).

### Cell proliferation, transwell, tube formation and wound closure assays

For analysing cell proliferation using the CCK-8 kit (BBI, e606336), we seeded 5000 cells/100 μL complete medium in a 96-well plate (Corning, 4515) and measured the absorbance at 450 nm every 12 h.

For the transwell assay, we seeded 5 × 10^4^ cells/200 μL basic medium onto the upper chamber in a 96-well plate and added 500 μL complete medium to the lower chamber. After 20 h, the upper chamber was removed to fix the samples and capture photographs.

For the tube formation assay, a mixture containing 50 μL BD Matrigel (Corning, 354234) and 50 μL medium was coated onto the 96-well plate, and the plate was incubated at 37 °C for 30 min. Next, HUVECs (3 × 10^4^ cells) were seeded into the wells of the plate and incubated for 6 h. The number of test tubes was measured using a microscope (Zeiss, Göttingen, German, Axio Obse) to determine the test tube formation ability. Each experiment was performed in triplicate.

For the wound closure assay, the cells were cultured in a six-well plate(Corning, 3516) to near confluence, and the culture medium was replaced with a serum-free medium. The dense cell layer was scraped with a sterile 200 mL pipette tip and starved for 48 h. Images were captured at 0 or 48 h.

### Exosome isolation and uptake

To isolate exosomes from the cellular supernatant, culture supernatant was prepared from a 48 h culture of CNE2 cells and then centrifuged by differential centrifugation at 500 × *g* for 10 min, 3000 × *g* for 60 min and 10,000 × *g* for 60 min. After filtering with a 0.22 m filter, the supernatant was transferred to an ultracentrifuge tube. Next, the supernatant was centrifuged by ultracentrifugation at 1,00,000 × *g* (XPN-100, Beckman Coulter) for 90 min at 4 °C. The exosome pellet was washed in ice-cold PBS and again ultracentrifuged. Finally, the isolated exosomes were resuspended in PBS.

To detect the uptake of exosomes by recipient cells, we labelled the exosomes using a PKH-26 labelling kit (Sigma-Aldrich, MINI26-1KT). Next, exosomes were cocultured with HUVECs for 2 h. We then fixed the cells with 4% paraformaldehyde and stained the nuclei with Hoechst. Finally, we obtained the images using a confocal microscope (Zeiss, Göttingen, German, Axio Obse).

In addition, in order to detect the uptake of exosomal HMGB3 by recipient cells, we used the upper and lower chamber coculture system. The upper chamber, containing a 0.4-μm pore-size filter (Corning, 353095), was planted with 5 × 10^4^ LV-HMGB3-GFP CNE2 cells, and the lower chamber, containing a six-well plate, was planted with the same amount of HUVECs. After 48 h, the nuclei of cells in the lower chamber were stained with Hoechst stain and photographed.

### Nanoparticle tracking analysis

The size and number of exosomes were detected using NanoSight (Malvern, NS300). A 488 nm laser camera was equipped to track Brownian motion and particle size for 60 s to produce a video. The videos were analysed using the NTA2.3 software.

### Transmission electron microscopy

First, 20 μL of fresh exosomes was dried in a copper grid for 15 min. Then, the samples were fixed in 2.5% glutaraldehyde for 1 h and stained with 3% phosphotungstic acid for 1 min. Finally, the exosomes were observed immediately using a transmission electron microscope (JEOL Ltd, Tokyo, Japan, JEM-1230).

### Flow cytometry analysis of exosomes isolated from clinical specimens

Exosomes were isolated from the sera of healthy individuals and patients with NPC and resuspended in PBS. Exosomes were transferred in a sterile 1.5 mL Eppendorf tube, stained with CellMask Green Plasma Membrane (CMG) (1:50; cat:C37608, Thermo Fisher Scientific, USA), and incubated in the dark for 30 min at 37 °C. Subsequently, exosomes were washed with 5 mL PBS to remove the excess CMG, and centrifuged at 1,00,000×*g* for 2 h at 4 °C. The supernatant was discarded and exosomes were lightly suspended in 100–150 μL PBS. For antibody staining of exosomes, the CMG-stained exosomes were incubated with anti-HMGB3 (cat:ab75782, Abcam, USA) conjugated with Alexa Fluor 594 at room temperature for 1 h and stirred gently in the dark. After 1 h, exosomes were washed in 5 mL PBS and centrifuged at 10,000×*g* for 2 h at 4 °C. The exosomes were then resuspended in 100–150 μL of PBS. To stain dsDNA in the CMG-labelled exosomes, DRAQ5 (1:50; cat:62254, Thermo Fisher Scientific, USA) was used, and the samples were incubated at room temperature for 1 h in the dark.

Furthermore, flow cytometry analysis using the Invitrogen Flow Cytometry Sub-micron Particle Size Reference Beads (cat:F13839, Thermo Fisher Scientific, USA) was performed to estimate the exosome particle size better, allowing the exclusion of those particles detected based on the signals from side scattering ≤150 nm.

### Matrigel plug angiogenesis assay

Five-week-old male nude mice were subcutaneously injected with a mixture of 300 μL HUVECs (3 × 10^6^ cells) and 300 μL of Matrigel containing 10 μg of exosomes. After 1 week, the plugs were obtained, fixed, sliced and stained with haematoxylin and eosin.

### In vivo analyses using zebrafish animal model

In vivo experiments, the estimated sample size can effectively detect significant differences between groups. The zebrafish and mice were randomly divided into two groups: the CNE2-NC group and the CNE2-shHMGB3 group. Investigators were blinded to the group allocation during the experiment.

NPC tumour cells were stained with DiI (Invitrogen, V-22885) for 30 min and then digested. Subsequently, the cells were washed twice in PBS and resuspended in Matrigel at a concentration of 4 × 10^6^ cells/30 μL. Approximately 10 nL of the mixed suspension containing the tumour cells, with or without knockdown of HMGB3, and Matrigel were injected into the perivitelline space of tg(kdrl: GFP) zebrafish embryos at 48 h post fertilisation (hpf)^28,29^. At 72 and 120 hpf, embryos were observed and photographed under a microscope (WPI, Sarasota, Florida, USA).

### Generation of orthotopic xenografts

For a mixed-cell injection, 4-week-old male nude mice were subcutaneously injected with a mixture of CNE2 cells (1 × 10^6^ cells) and an equal amount of HUVECs in 200 μL of Matrigel:PBS in a 1:1 ratio. After 7 days, the xenografts were harvested, fixed, sliced and stained.

For proliferation experiments, 4-week-old male nude mice were subcutaneously injected with CNE2 cells (1 × 10^6^ cells) with or without HMGB3 knockdown. After 2 weeks, the xenografts were harvested, fixed, sliced and stained.

### Immunohistochemistry (IHC)

Tissue-embedded sections were washed repeatedly in xylene (three times), 100% ethanol (two times), 70% ethanol (two times) and water (two times) for 10 min. The sections were then boiled in sodium citrate buffer for 1 h. The peroxidase blocker was added drop wise for 10 min, and tissues were incubated with the primary antibody at 4 °C overnight. The primary antibodies used were anti-CD34 (cat:14486-1-AP, Proteintech, China, 1:100) and anti-HMGB3 (cat:D160490, Sangon Biotech, China, 1:50). The reaction enhancer and the secondary antibody were added drop wise on the following day, and tissues were incubated for 1 h at room temperature. HMGB3 and CD34 expression were observed using 3,3′-diaminobenzidine, and samples were stained with hematoxylin and eosin. In brief, we measured the MVD, by dividing each xenograft into three layers and cutting three tissue slices from each layer. MVD was evaluated as previously described by Foote^30^. We found areas with a high MVD under a low magnification (40×). Then, single microvessels were counted in three fields under a magnification of 200× or 400×.

### Statistical analysis

Statistical analyses were conducted using GraphPad Prism 8 (GraphPad Software, San Diego, CA, USA) and IBM SPSS Statistics version 21 (IBM Corp., Armonk, NY, USA). The data are presented as the mean ± standard error of the mean and were analysed using one-way ANOVA and Student’s *t*-test. Correlation analysis was performed using Spearman’s rank correlation coefficient. Survival analysis was analyzed using Kaplan–Meier survival curves. The sample size of all experiments was determined based on our experience. No samples were excluded from the analysis. All animals were randomly assigned for the experiments. All experiments were conducted with three independent replicates. The following *P* values denoted statistical significance: *P < 0.05, **P < 0.01, ***P < 0.001 and ****P < 0.001.

## Results

### HMGB3 upregulation is associated with NPC progression

**Fig. 1:**
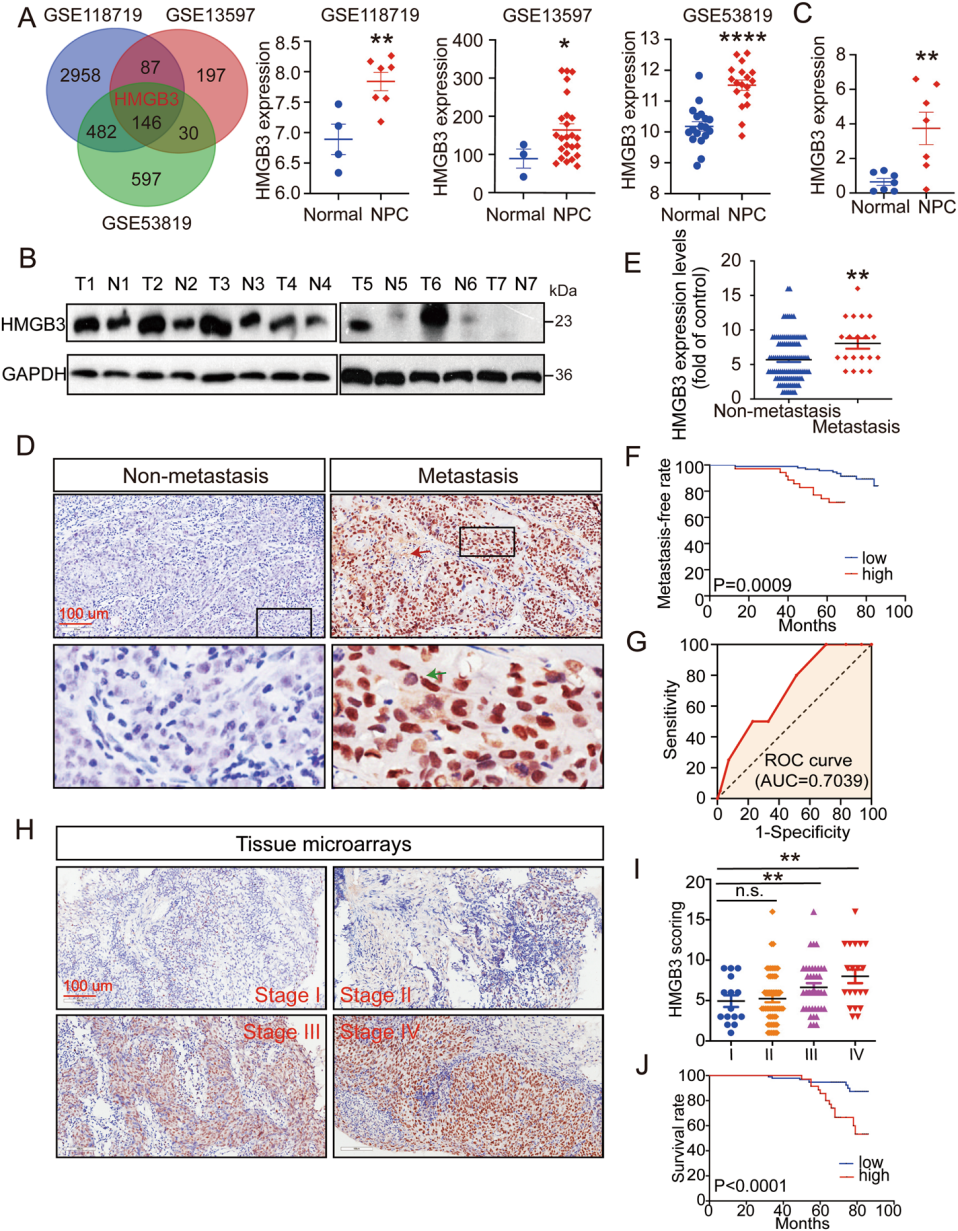
Overexpression of HMGB3 is related to *clinical outcomes* of NPC patients. **A** A Venn diagram of all upregulated genes among three GEO databases (GSE118719, GSE13597 and GSE53819) and the expression of HMGB3 in them. **B** HMGB3 protein levels in NPC tissues (*n* = 7) and normal nasopharyngeal epithelial tissue (*n* = 7) were examined by western blot. GD(GAPDH) was used as a loading control. **C** HMGB3 mRNA levels in NPC tissues (*n* = 7) and normal nasopharyngeal epithelial tissue (*n* = 7) were examined by qRT-PCR. **D** Representative IHC images of HMGB3 staining from NPC patients with or without metastasis and the black boxes indicate regions of pictures shown in the bottom two pictures. **E** Statistical comparison of HMGB3 score in the two groups. **F** The HMGB3 score was defined as low expression (1–8 points)(*n* = 94) and high expression (9–16 points)(*n* = 35). And the Kaplan–Meier analysis in the relationship between HMGB3 expression and metastasis of NPC. **G** The receiver operating characteristic (ROC) curve was plotted to validate the predictive accuracy of HMGB3 expression in metastasis NPC (ROC AUC = 0.7039). **H** Representative IHC images of HMGB3 staining in four TNM stages. **I** Statistical comparison of HMGB3 score in the four groups. **J** Kapla–Meier survival analysis of NPC patients’ overall survival by X-tile Software. The scale bar in 200× images represents 100 µm. Mean ± SD, *P < 0.05, **P < 0.01, ***P < 0.001, ****P < 0.0001, student’s test.

Presently, many studies have reported that the abnormal expression of certain genes is closely related to tumour malignancy, metastasis, and patient survival. To identify genes that are abnormally expressed in NPC, we utilised three Gene Expression Omnibus (GEO) datasets (GEO accession numbers: GSE13597, GSE53819 and GSE118719), the intersection of which revealed 146 upregulated genes (*p* < 0.05 and logFC > 0.8) (Fig. 1A). The main localisation of these genes is summarised in Supplementary Table 1. Since the present research was dedicated to nEXOs, which are confirmed to be secreted by cancer cells in amounts higher than that by normal cells^19^, we focused on the genes mainly located in the nucleus and extracellular matrix. Consequently, four genes, HOXA10, LHX2, GADD45A and HMGB3, were selected. In addition, we used the HNSCC data from The Cancer Genome Atlas (TCGA) to detect the expression of these four genes (http://gepia.cancer-pku.cn). The data showed that HMGB3 and HOXA10 were significantly upregulated in HNSCCs (Supplementary Fig. 1A). However, HOXA10 has been reported in NPC^31^. Therefore, we aimed to study the role of HMGB3 in NPC. As demonstrated in the three datasets, the western blot and qRT-PCR analyses showed that HMGB3 expression was significantly increased in NPC tissues compared with that in the normal nasopharyngeal tissues (Fig. 1B, C). To gain further insight into the clinical significance of HMGB3, IHC for NPC tissue microarrays (*n* = 129) was performed, along with HMGB3 expression analysis. The staining intensity and the positive staining rate were both categorised into four grades (scored from 1 to 4 points), the product of which formed a total score. As shown in Fig. 1D–G, there was a significant positive correlation between HMGB3 upregulation and distant metastasis. Based on the score and TNM stages, the results showed that HMGB3 expression in patients at stages III and IV was higher than that in patients at stage I (Fig. 1H, I). Furthermore, increases in HMGB3 expression paralleled an increase in NPC recurrence (*P* = 0.008, Supplementary Table 2). The Kaplan–Meier survival curves showed that high HMGB3 expression was associated with poor survival outcomes in patients with NPC, as observed using X-tile Software (Fig. 1J). These data suggested that HMGB3 was highly expressed and affected clinical outcomes in NPC.

### Increases in extracellular HMGB3 expression and micronuclei numbers are relevant to NPC progression

**Fig. 2:**
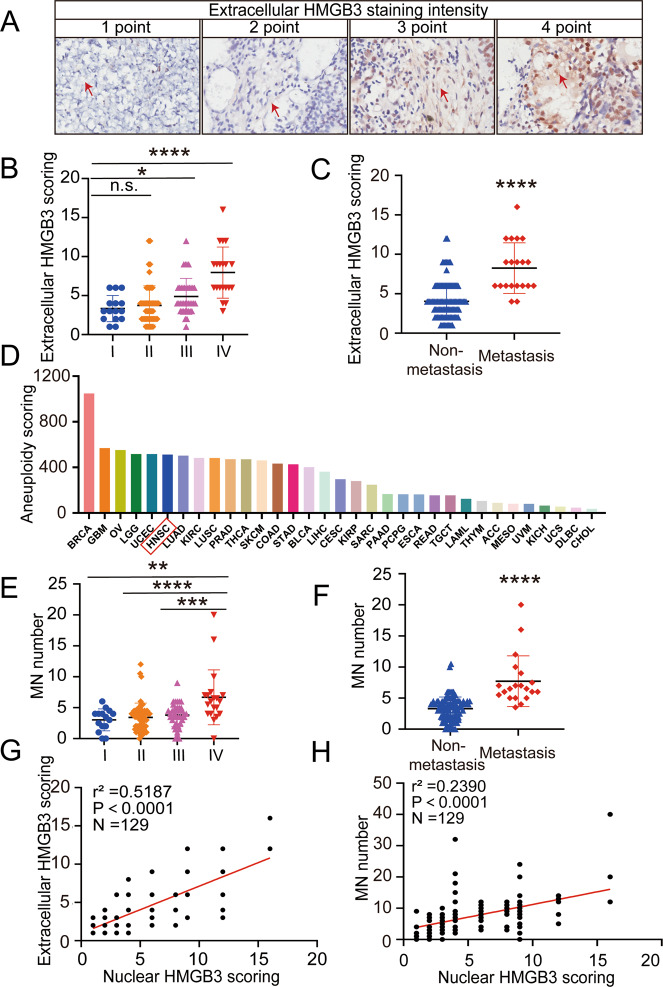
The relationship of nuclear HMGB3, extracellular HMGB3 and MN numbers. **A** Representative IHC images of extracellular HMGB3 staining and the red arrow points to part of the extracellular staining area. **B** Statistical comparison of extracellular HMGB3 score in TNM stages. **C** Statistical comparison of extracellular HMGB3 score in the two groups with or without metastasis. **D** The pan-cancer analysis of the chromosome aneuploidy. **E** Statistical comparison of the MN numbers in TNM stages. **F** Statistical comparison of the MN numbers in the two groups with or without metastasis. **G** Spearman correlation between nuclear HMGB3 score and extracellular HMGB3 score. **H** Spearman correlation between nuclear HMGB3 score and the MN numbers. Pearson correlation coefficient (*r*^2^) and *P* value are shown. Mean ± SD, *P < 0.05, **P < 0.01, ***P < 0.001, ****P < 0.0001, student’s test.

We found that the nuclear protein HMGB3 was also expressed in the extracellular matrix (red arrow) (Fig. 1D). Subsequently, we analysed the correlation between the expression of HMGB3 in the extracellular matrix and NPC progression. First, we categorised the staining intensity of the extracellular matrix HMGB3 into four levels (scored from 1 to 4 points) (Fig. 2A). Next, the positive staining rate was classified into four grades (scored from 1 to 4 points) according to the percentage of the total extracellular matrix area: 0–25% is 1 point, 25–50% is 2 points, 50–75% is 3 points and 75–100% is 4 points. The product of the two provided the final score. Compared with stage I, stages III and IV were associated with higher expression of extracellular HMGB3 (Fig. 2B). We also evaluated the extracellular matrix expression of HMGB3 in NPC tissues and found that it was significantly higher in metastatic tissues than in non-metastatic NPC tissues (Fig. 2C). Interestingly, HMGB3 is micronuclei specific protein^32^ and micronuclei were detected in the tissue chip (green arrow) (Fig. 1D). Besides, the upregulation of HMGB1, homologous gene of HMGB3, leads to genome instability^33^. Since the instability of the genome is closely related to micronuclei production, we analysed the chromosome aneuploidy scores using the pan-cancer database^20^. The data showed that HNSCC had a higher incidence of aneuploidy (Fig. 2D). Similarly, compared with stage I, II and III NPC tissues, the micronuclei number in stage IV NPC tissues was significantly increased (Fig. 2E). In addition, IHC showed a significant increase in micronuclei numbers in the metastatic NPC tissues (Fig. 2F). Moreover, high levels of nuclear HMGB3 correlated positively with high levels of extracellular HMGB3 and micronuclei formation (Fig. 2G, H). In summary, a synergy of nuclear HMGB3 expression, extracellular HMGB3 expression and micronuclei production also affected NPC progression.

### HMGB3 regulates NPC cell proliferation and migration in vitro

**Fig. 3:**
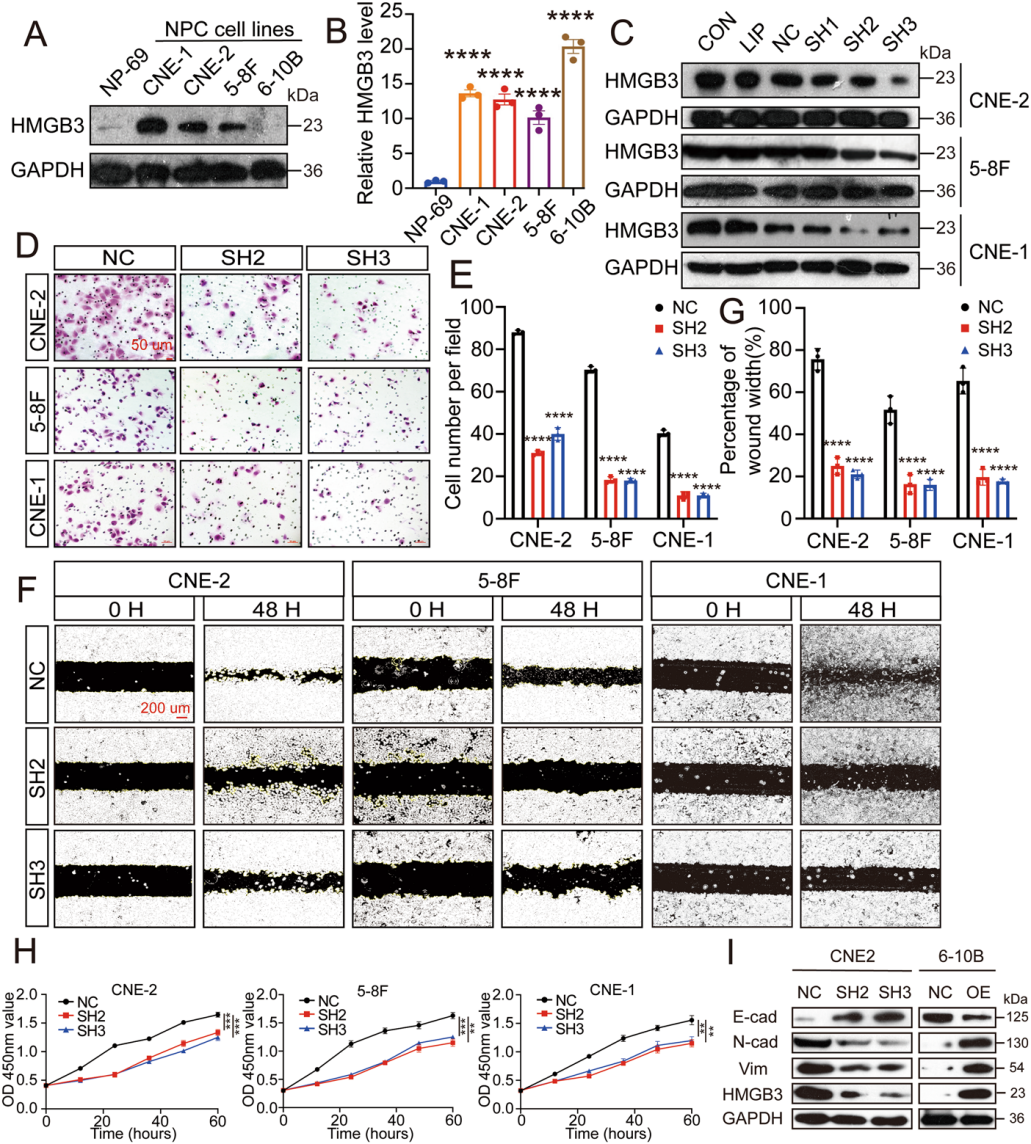
HMGB3 knockdown reduces NPC cell proliferation and migration in vitro. **A** HMGB3 protein levels in NP69 and NPC cell lines (CNE1, CNE2, 5–8 F and 6–10B) were determined by western blot. **B** HMGB3 mRNA levels in NP69 and NPC cell lines were examined by qRT-PCR (one-way ANOVA). **C** Transfection efficiency of three NPC lines (CNE1, CNE2 and 5–8 F) was measured by western blot. CON control, LIP lip2000, NC negative control, SH1 shHMGB3-1, SH2 shHMGB3-2, SH3 shHMGB3-3. **D** The transwell assay was performed to measure cell migration. The scale bar represents 50 µm. **E** Statistical comparison of the cell number of the transwell assay in the two groups. **F** The wound closure assay was performed to measure cell migration. The scale bar represents 200 µm. **G** Statistical comparison of the percentage of wound width in the two groups. **H** The CCK8 assays were performed to measure cell proliferation. All experiments were conducted with three independent replicates. **I** The levels of HMGB3 and EMT markers in NPC cell lines after knockdown or overexpression of HMGB3 were determined by western blotting. GAPDH expression was used as a loading control. Mean ± SD, **P < 0.01, ***P < 0.001,****P < 0.0001, student’s test.

The subsequent experiments demonstrated the HMGB3 expression in the NPC cell lines (CNE1, CNE2, 5–8 F and 6–10B) and normal nasopharyngeal epithelial cell line NP69. Western blot and qRT-PCR analyses showed that HMGB3 expression was dramatically increased in the NPC cell lines, except 6–10B, for which high expression was noted only in western blotting (Fig. 3A, B). We also detected the expression of other genes in the HMGB family (HMGB1, HMGB2 and HMGB4) (Supplementary Fig. 1B). Finally, we chose CNE2, CNE1 and 5–8 F cell lines for the subsequent analyses. To investigate the role of HMGB3 in the malignant progression of NPC, three HMGB3 knockout plasmids were used. The transfection efficiencies of the plasmids were detected by western blotting in these three NPC cell lines. The effective plasmids, shHMGB3-2 and shHMGB3-3, were selected for the following experiments (Fig. 3C). Transwell and wound closure assays (Fig. 3D–G) showed that HMGB3 knockdown inhibited the migration ability of the three NPC cell lines. Moreover, a similar trend was observed in the proliferation ability by the CCK-8 assay (Fig. 3H). Additionally, to determine whether the ectopic expression of HMGB3 facilitated the NPC cells with increasing malignant properties in vitro, we selected a low-HMGB3-expression cell line 6–10B by transfecting with ectopic HMGB3. As expected, enhancements of cell proliferation and migration ability were observed in 6–10B oeHMGB3 compared with the 6–10B negative control (Supplementary Fig. 2A–F). Epithelial-mesenchymal transition (EMT) has been involved in cancer progression. To investigate the regulatory effect of HMGB3 on cell EMT, we analyzed the expression of EMT markers. As illustrated in Fig. 3I, the expression of epithelial-associated marker (E-cadherin) was increased while mesenchymal-associated markers (vimentin and N-cadherin) were decreased after HMGB3 knockdown in CNE2 cells. Likewise, HMGB3 overexpression in 6–10B cells show the same result (Fig. 3I). These observations indicated that HMGB3 regulated the metastasis, proliferation and EMT of NPC cells in vitro.

### HMGB3 positively correlates with the MVD

**Fig. 4:**
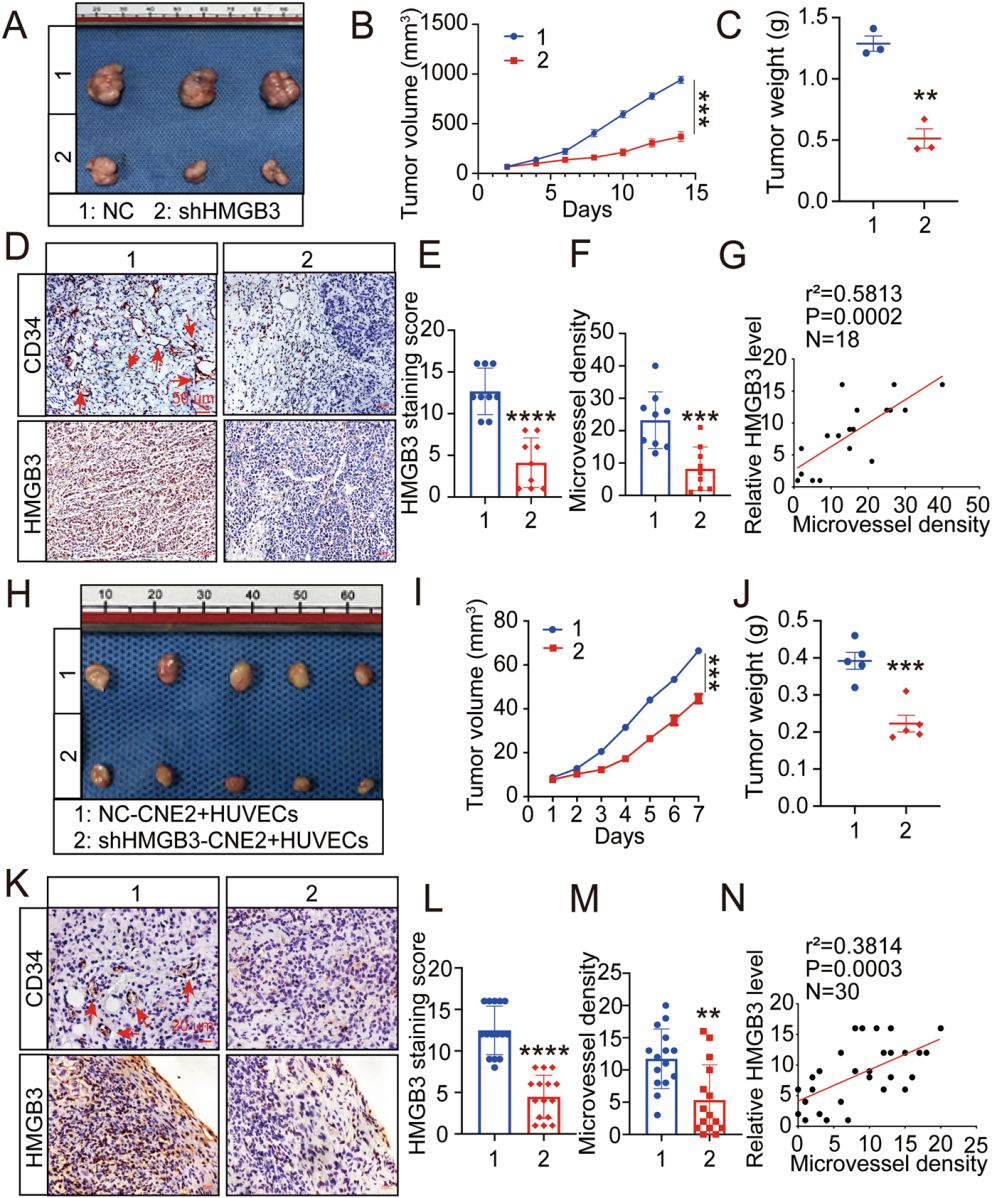
Overexpression of HMGB3 correlates positively with MVD in NPC xenografts. **A**, **H** Images of corresponding tumour xenografts. **B**, **I** Tumour volume in the two groups. **C**, **J** The weights of the excised xenografts. **D**, **K** Representative images of IHC for CD34 and HMGB3 in tissues collected from two groups of NPC xenografts. **E**, **L** Quantification of IHC staining score for HMGB3 expression. **F**, **M** Statistics of microvessel density. **G**, **N** Spearman correlation between HMGB3 score and MVD in tumour xenografts. Pearson correlation coefficient (*r*^2^) and *P* value are shown. The scale bar in 400× images represents 50 µm. The red arrows indicate microvessels. Mean ± SD, **P < 0.01, ***P < 0.001, ****P < 0.0001, student’s test.

**Fig. 5:**
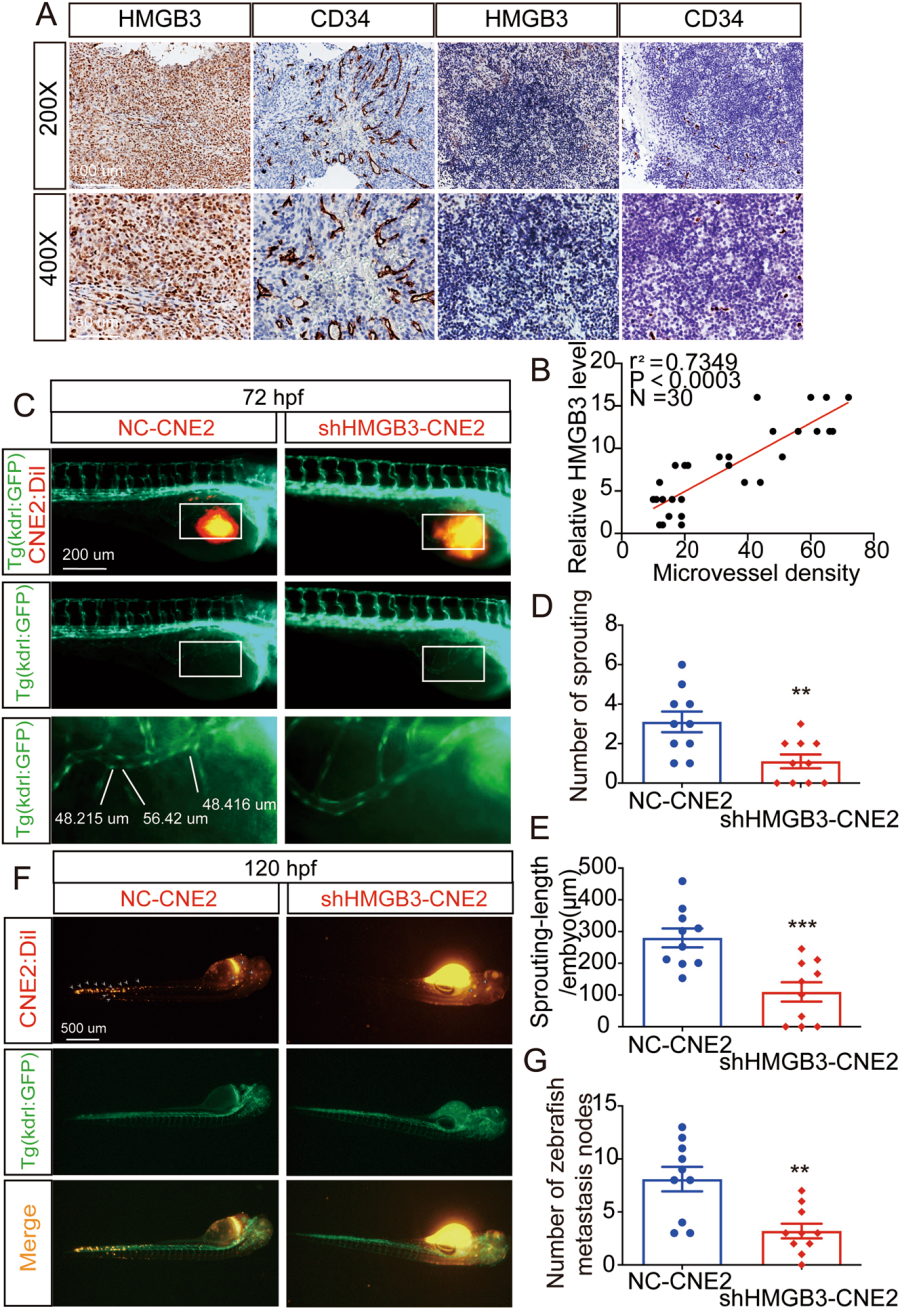
The positive correlation between HMGB3 and MVD in NPC patients and the further study that CNE2 with HMGB3 knockdown inhibited tumour angiogenesis and metastasis in tg(kdrl:GFP) zebrafish embryos. **A** Representative images of IHC for CD34 and HMGB3 in NPC tissues. The scale bar in 200× images represents 100 µm. **B** Spearman correlation between HMGB3 score and MVD in NPC patients. Pearson correlation coefficient (*r*^2^) and *P* value are shown. **C** The overall shape of 96 hpf embryos after injection of about 10 nL of Matrigel/CNE2-NC or Matrigel/CNE2-shHMGB3 solution in the perivitelline space at 48 hpf. White boxes indicate regions of pictures shown in the bottom two pictures and the analysis of the subintestinal venous plexus (SIV) was conducted. The scale bar represents 200 µm. **D** Quantifications with the number of sprouting of ten embryos per group. **E** Quantifications with the sprouting-length per embryo of ten embryos per group. **F** At 120 hpf, the migration of CNE2 cells was measured using fluorescence microscopy. The scale bar represents 500 µm. **G** Quantification of migratory cell numbers of ten embryos per group. Mean ± SD, **P < 0.01, ***P < 0.001, student’s test.

Based on the effects of HMGB3 on CNE2 cell proliferation observed in vitro, we further conducted in vivo analysis using 4-week-old nude mice. We injected CNE2-NC or CNE2-shHMGB3 (plasmid shHMGB3-3) subcutaneously into nude mice. Two weeks later, the xenografts were excised and weighed (Fig. 4A). Compared with the CNE2-NC group, the CNE2-shHMGB3 group exhibited slower growth (Fig. 4B) and had lesser weight (Fig. 4C). Of note, on the surface of CNE2-shHMGB3 xenografts, several small blood vessels were observed. We measured the MVD of these new blood vessels. To investigate the relationship between HMGB3 and MVD, IHC was performed to detect HMGB3 and CD34 expression (Fig. 4D). Consequently, the Spearman correlation analysis showed that HMGB3 expression levels were positively correlated with the MVD (Fig. 4E–G). However, subcutaneous tumour formation recruited a large number of blood vessels in nude mice. For the sake of human tumour angiogenesis, CNE2 cells and HUVECs were mixed in Matrigel and injected subcutaneously in 4-week-old nude mice. One week later, the xenografts were excised and assessed (Fig. 4H–J). Consistently, high HMGB3 expression was significantly correlated with the increased MVD (Fig. 4K–N). To verify the relationship between HMGB3 and the MVD in clinical samples, 30 NPC tissue samples were probed with HMGB3 and CD34 antibodies (Fig. 5A). IHC indicated a positive correlation between HMGB3 and the MVD (Fig. 5B). Taken together, these findings provided evidence that HMGB3 in NPC could positively regulate angiogenesis.

### HMGB3-deficient NPC cells affect angiogenesis and metastasis in zebrafish

To investigate whether HMGB3-induced angiogenesis and tumour metastasis are linked, we further established tumour xenografts in zebrafish embryos. At 72 hpf, we observed ectopic angiogenesis in the subintestinal vein (Fig. 5C). Compared with the CNE2-NC cells, the CNE2-shHMGB3 cells showed a decrease in the number of sprouts and sprouting length per embryo (Fig. 5D, E). Subsequently, on the third day post injection, we found that there were greater number of metastatic tumour cells in the tail and head of the zebrafish in the CNE2-NC group, than that observed on the day of injection (Fig. 5F, G). It is worth noting that, excluding the changes in the migration ability of tumour cells, new blood vessels may promote tumour metastasis by providing metastatic ducts. In summary, in a zebrafish model, HMGB3 could regulate angiogenesis and promote tumour metastasis.

### CNE2 cell-secreted HMGB3 is transferred to HUVECs via nEXOs

**Fig. 6:**
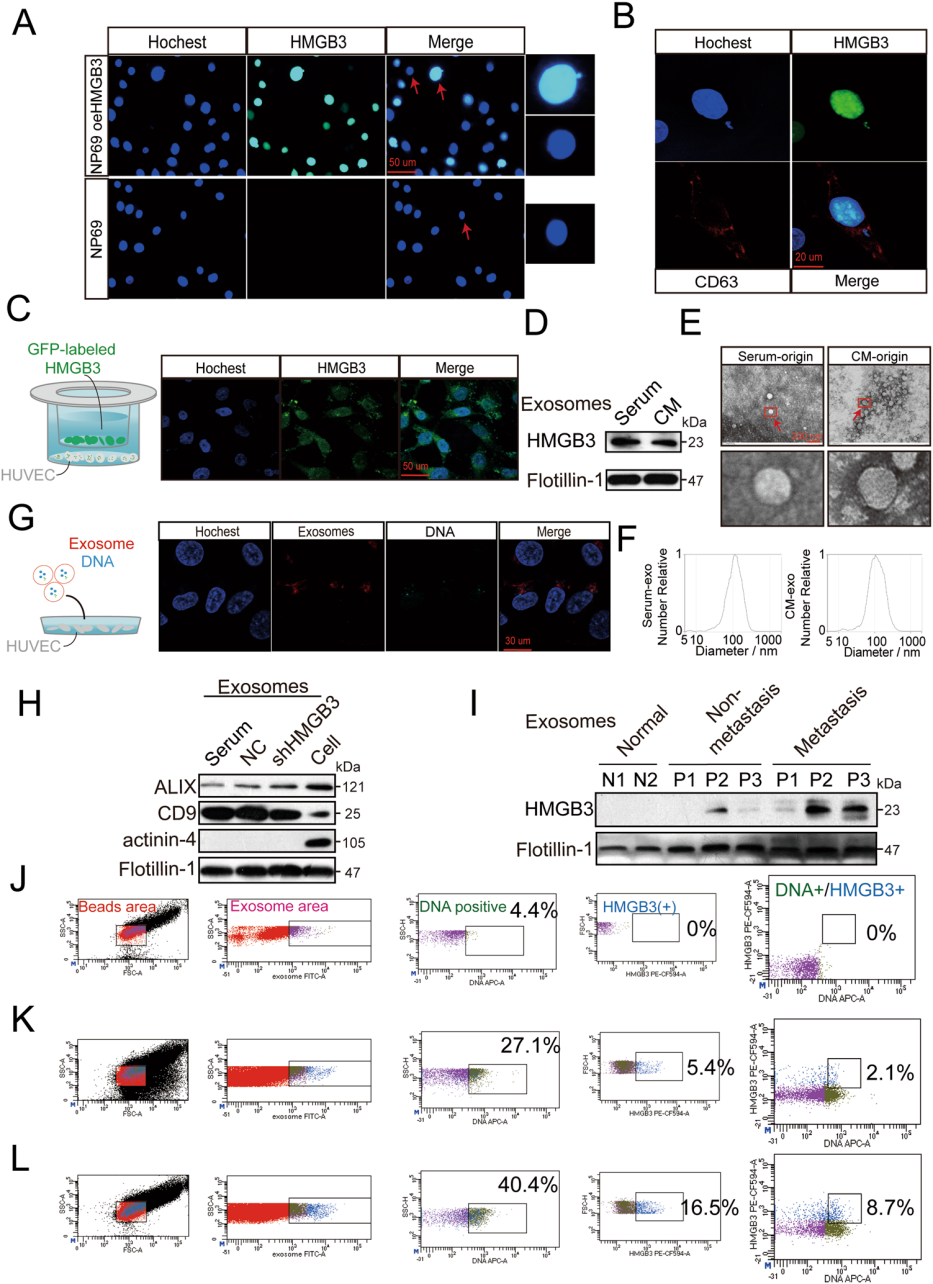
Nuclear exosomes HMGB3 derived from the micronucleus can be transferred from CNE2 to HUVECs and is associated with NPC metastasis. **A** Immunofluorescence (IF) images of NP69 with or without HMGB3 overexpression. The right images show a magnified nucleus and MN. The scale bar represents 50 µm. **B** IF images of NP69 oeHMGB3 were stained by HMGB3 (green), CD63 (red), and nucleus (blue). The scale bar represents 20 µm. **C** CNE2 cells transfected with fluorescent GFP-labelled HMGB3 overexpresses lentivirus were cocultured with HUVECs in a transwell Chamber. The scale bar represents 50 μm. **D** Western blotting analysis of the expression of HMGB3 in exosomes from NPC serum and CNE2-CM (cell medium). **E** Transmission electron microscopy of exosomes derived from NPC serum and CNE2-CM. The scale bar represents 200 nm. The red boxes indicate regions of pictures shown in the bottom two pictures. **F** Nanoparticle tracking analysis showed the size and distribution of exosomes isolated from NPC serum and CNE2-CM. **G** HUVECs uptake of exosomes released by NPC with a confocal microscope. blue: Hoechst staining; red: PKH26-labelled exosomes; light blue: DRAQ5 dye-labelled DNA. **H** Western blotting analysis of Alix, CD9, actinin-4 and flotillin-1 in NPC exosomes and CNE2 cells. **I** Detection of HMGB3 in serum exosomes of NPC patients and normal persons by Western blot. **J**–**L** Flow cytometry analysis showed the percentage of gDNA and HMGB3 expression in exosomes of NPC patients and normal persons. J: normal persons; K: NPC patients without metastasis; L: NPC patients with metastasis.

Tissue microarray analysis showed a positive correlation between HMGB3 expression and micronuclei formation (Fig. 2G, H), which can produce nEXOs^19^. Moreover, HMGB3 expression in NPC cells affected angiogenesis. Based on these findings, we investigated whether HMGB3 could be taken up by HUVECs in the form of nEXOs. First, compared with NP69 cells, NP69 oeHMGB3 cells promoted the production of micronuclei (Fig. 6A). The micronuclei, also HMGB3-positive, could be surrounded by CD63, which is involved in exosome cargo loading (Fig. 6B). We transfected CNE2 cells with fluorescent GFP-labelled HMGB3 and assessed the GFP level by fluorescence microscopy after 3 days of coculturing with HUVECs in the upper and lower chambers. Green fluorescence appeared in HUVECs, indicating that GFP-HMGB3 could be taken up by HUVECs from CNE2 cells (Fig. 6C). To detect the secretion of exosomes, we first separated the exosomes from the CNE2 cell culture medium (CM) and serum of patients with NPC by ultracentrifugation. Western blot analysis showed that these two exosomes contained HMGB3 (Fig. 6D). Subsequently, transmission electron microscopy and nanoparticle tracking analysis were used to analyse the purified exosomes. As shown in Fig. 6E,F, exosomes are lipid bilayer membranes with an average diameter of 108 nm (serum-exo) or 110.4 nm (CM-exo). Furthermore, the exosome markers Alix, CD9 and flotillin-1 were highly abundant in the exosomes isolated, thereby confirming their purity. Actinin-4 was hardly detectable in the exosomes, but was abundant in the cells, indicating that the exosomes isolated were relatively pure and not contaminated by other cell organelles or impurities (Fig. 6H). Subsequently, we investigated whether nEXO HMGB3 could be taken up by HUVECs. Exosomes with a high HMGB3 expression were labelled with the red fluorescent dye PKH-26 and stained with DNA markers. The prepared exosomes were cocultured with recipient cells (HUVECs). After 24 h, exosomes and DNA were found to be surrounding the nucleus of HUVECs. This indicated that the nEXOs containing HMGB3 could be transferred from CNE2 cells to HUVECs (Fig. 6G). To quantify and evaluate the expression levels of circulating nEXO HMGB3, exosomes from patients with NPC and healthy individuals were isolated. We found that nEXO HMGB3 was greatly increased in patients with NPC, especially in the metastatic patients, compared with that in the healthy individuals (Fig. 6I). Analogously, flow cytometry analysis was used to quantify the population of exosomes, which indicated that the metastatic patients secreted a greater amount of nEXOs containing HMGB3 than that by the non-metastatic patients. However, we could not detect nEXOs containing HMGB3 in the serum of healthy individuals (Fig. 6J–L). Collectively, these results demonstrated that nEXO HMGB3 could be efficiently taken up by HUVECs and was related to NPC metastasis.

### nEXO HMGB3 promotes metastasis by inducing angiogenesis

**Fig. 7:**
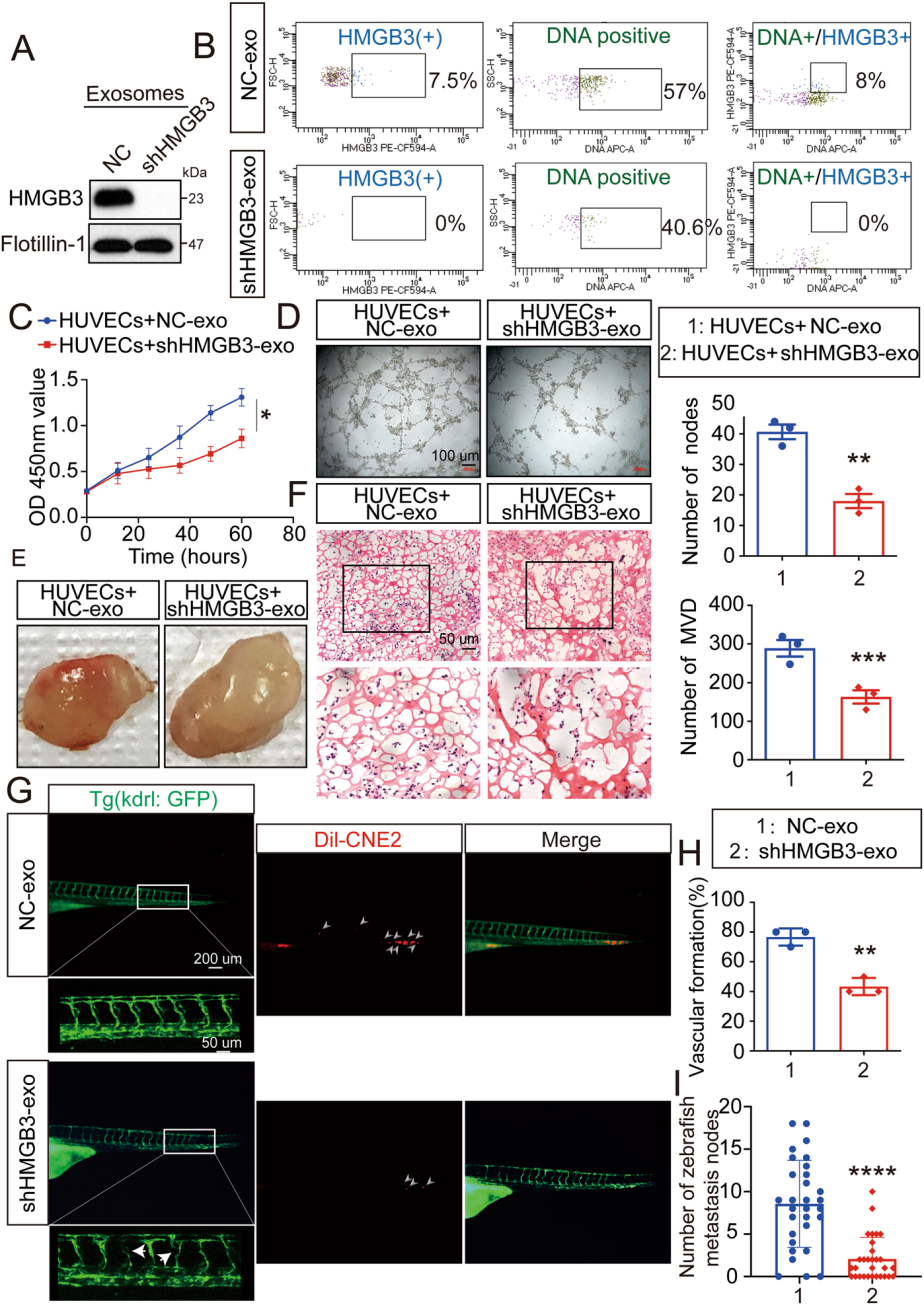
nEXOs HMGB3 regulates metastasis via angiogenesis. **A**The HMGB3 levels of CNE2-NC-exo and CNE2-shHMGB3-exo were measured by western blot. **B** Flow cytometry analysis showed the percentage of gDNA and HMGB3 expression in exosomes of CNE2 cell supernatant. **C** The CCK8 assays were performed to measure proliferation of HUVECs treated with the two exosomes. **D** The tube formation assays were performed to measure tube forming ability of HUVECs treated with various exosomes. The scale bar represents 200 μm. **E** Gross-observation exosomes modulated angiogenesis. **F** Frozen slices of the Matrigel plugs were stained with eosin-hematoxylin. The scale bar in 200× images represents 100 µm. The scale bar in 400× images represents 50 µm. **G** The GFP‐positive (green) intersegmental vessel (ISV) and CNE2 cells (red) are photographed by a microscope for 30 embryos per group. The red box was further photographed with a confocal microscope. The red arrow indicates a vascular defect. White arrows indicate metastasis nodes. **H** Percentage of vascular formation of ten embryos per group. **I** Quantification of migratory cell numbers. All experiments were conducted with three independent replicates. Mean ± SD, **P < 0.05, **P < 0.01, ***P < 0.001, student’s test.

To analyze the relationship between metastasis-related nEXO HMGB3 and angiogenesis, a coculture model was established. First, CNE2 cells were infected with shHMGB3 plasmids (shHMGB3-3) or NC control vector plasmids. Next, we collected the cell supernatant at 48 h and isolated NC-containing (NC-exo) or low-HMGB3-containing (shHMGB3-exo) nEXOs. Furthermore, we confirmed the expression of HMGB3 in both exosomes by western blotting (Fig. 7A). Importantly, flow cytometry analysis showed that CNE2 cells knocked down HMGB3 did not secrete nEXO containing HMGB3. Moreover, knocking down HMGB3 can inhibit the production of micronuclei and further reduce the secretion of nEXO (Fig. 7B). Subsequently, further studies were needed to determine whether nEXO HMGB3 affected angiogenesis and metastasis. Compared with coculturing with NC-exo, HUVECs cocultured with shHMGB3-exo inhibited angiogenesis-related processes in vitro, including proliferation (Fig. 7C) and tube formation (Fig. 7D). Besides, we performed a matrigel plug angiogenesis assay in vivo. Compared with NC-exo, HUVEC plugs containing shHMGB3-exo exhibited lesser angiogenesis, suggesting that a lack of nEXO HMGB3 reduced angiogenesis (Fig. 7E, F). To gain further insight into the involvement of nEXO HMGB3 in angiogenesis and metastasis, the two exosomes were added to the E3 solution to culture tg(kdrl: GFP) zebrafish embryos at 8 hpf. Then, we injected Dil-CNE2 into the perivitelline space of tg(kdrl: GFP) zebrafish at 48 hpf. At 120 hpf, we found that the shHMGB3-exo group had lesser vascular formation and metastasis nodes than NC-exo group (Fig. 7G). In brief, these data provided evidence that nEXO HMGB3 could significantly promote metastasis by inducing angiogenesis.

## Discussion

Research over the past decade has established that tumour growth and metastasis depend on angiogenesis, which provides tumour cells with sufficient oxygen and nutrients. Therefore, drugs aimed at reducing the oxygen and nutrient supply, namely angiogenesis inhibitors, are considered promising antitumour treatments^34,35^. Moreover, targeting tumour angiogenesis has been a widely accepted clinical treatment strategy in recent years. However, the development of tumours has not been significantly altered by existing anti-angiogenesis treatments, partly because of the incomplete suppression of tumour angiogenesis. Therefore, there is an urgent need to find new targets for anti-angiogenesis treatment. Tumours can affect angiogenesis in many ways, including secretion of pro-angiogenic factors, formation of a hypoxic environment, effects on other cells in the tumour microenvironment and secretion of exosomes. For example, VEGF secretion is promoted in ovarian cancer cells that overexpress OVA66^36^. Azoitei et al. ^37^ reported that in hypoxic pancreatic tumours, PKM2 promotes tumour angiogenesis through HIF-1α. Chen et al. ^38^ reported that the TSC2-mTOR pathway regulates macrophage-induced tumour angiogenesis. In our study, we found that in nude mice xenografts, increases in HMGB3 expression paralleled an increase in MVDs. Similar tendencies were observed in a zebrafish/tumour xenograft model, in which HMGB3 expression was significantly correlated with the sprouting of the subintestinal vein. Subsequent observation showed that greater sprout formation enhanced tumour cell metastasis. Notably, in our tissue samples, a high expression of HMGB3, increased angiogenesis, and patient tumour metastasis were positively correlated. Collectively, we confirmed that HMGB3 is involved in tumour angiogenesis. Interestingly, previous studies have shown that HMGB3 has binding sites for MIR-205^39^, which is a microRNA associated with HNSCC, and induces tumour angiogenesis in ovarian cancer^40^. Since then, it has been of great interest to study the relationship between HMGB3 and tumour angiogenesis.

Exosomes, as carriers of signal transmission, can transfer various biological molecules across cells, such as proteins, RNA and DNA. For example, our team previously demonstrated that metastasis-related MIR-23a promotes angiogenesis in NPC via exosomes^41^. Here, we particularly focused on nEXOs because HMGB3 is a nuclear protein. nEXOs are derived from micronuclei, and these serve as markers of genomic instability. nEXOs can almost only be detected in tumour exosomes, and not in normal cells. nEXOs can also be used as important biomarkers for cancer detection and facilitate the longitudinal monitoring of cancer patients^19^. Takahashi et al. ^42^ reported that nEXOs maintain cell homoeostasis by transporting harmful genetic DNA outside the cell. To our knowledge, the present study is the first study to analyse the role of a certain protein in nEXOs. First, we detected and quantified the expression of HMGB3 in nEXOs through western blotting and flow cytometry, and found that it was positively correlated with NPC metastasis. Therefore, these data suggested that HMGB3 levels in circulating nEXOs serve as valuable biomarkers of NPC metastasis. nEXOs can promote inflammation through the cGAS/STING pathway, which provides nutrients to the pre-metastatic niche^42,43^. In addition, chromosomal instability is closely associated with tumour metastasis^44^. Second, we found that HMGB3 in nEXOs could be taken up by HUVECs and stimulated angiogenesis, thereby promoting NPC metastasis. However, our study had a few limitations; it could not elucidate the mechanism underlying the high HMGB3 expression resulting in the production of micronuclei. HMGB3 can bind to DNA and bend it^45^. Mukherjee et al. ^46^ reported that HMGB3 promotes DNA damage through the ATR/CHK1/p-CHK1 signalling pathway. Moreover, micronuclei contain not only proteins, but also genomic DNA. Recently, it has been reported that the extrachromosomal DNA (ecDNA) is almost non-existent in normal cells and is closely related to tumour progression. Therefore, our next steps would be to study the relationship between HMGB3, micronuclei and ecDNA.

In conclusion, our findings revealed the role of HMGB3 towards the malignant phenotype of NPC, with HMGB3 expression being associated with NPC angiogenesis and metastasis. Importantly, nEXO HMGB3, originating from micronuclei, were mainly detected in metastatic NPC patients. Furthermore, nEXO HMGB3, secreted by NPC cells, promoted tumour metastasis by inducing angiogenesis. Therefore, we confirmed that nEXO HMGB3 could be a significant biomarker of NPC metastasis, thereby providing novel insights into the clinical application of anti-angiogenesis therapy for tumours.

## Supplementary information

Gene intersection of three GEO databases and the main localisation of these genes

Relationship between HMGB3 expression and clinicopathological characteristics of NPC patients

The expression of the four genes in the TCGA database and the expression in cell lines of the HMGB family.

HMGB3 overexpression promote NPC cell proliferation and migration in vitro.
