# Supplementary material for: Nuclear exosome HMGB3 secreted by nasopharyngeal carcinoma cells promotes tumour metastasis by inducing angiogenesis
Source: Cell Death Dis. 2021 May 28;12(6):554. doi: 10.1038/s41419-021-03845-y (PMC8163785; doi:10.1038/s41419-021-03845-y)
Supplement: Supplementary file 1 — Gene intersection of three GEO databases and the main localisation of these genes [file 41419_2021_3845_MOESM1_ESM.docx]

**Table S1. Gene intersection of three GEO databases and the main localization of these genes(https://www.genecards.org/).**

|  |  |  |  |
| --- | --- | --- | --- |
| Number | Gene | Localization |  |
| 1 | ABCA3 | plasma membrane | extracellular |
| 2 | GPSM2 | cytoskeleton | cytosol |
| 3 | COL4A5 | extracellular | endoplasmic reticulum |
| 4 | IGSF3 | plasma membrane | extracellular |
| 5 | BRCA1 | plasma membrane | cytoskeleton |
| 6 | LMNB2 | nucleus | cytoskeleton |
| 7 | PDCD5 | nucleus | cytosol |
| 8 | FOXM1 | nucleus | cytoskeleton |
| 9 | CDK1 | extracellular | cytoskeleton |
| 10 | PLXNA1 | plasma membrane | nucleus |
| 11 | COL7A1 | extracellular | endoplasmic reticulum |
| 12 | UNG | mitochondrion | nucleus |
| 13 | KIF14 | cytoskeleton | cytosol |
| 14 | C17orf53 | nucleus | cytosol |
| 15 | MAD2L1 | cytoskeleton | nucleus |
| 16 | FXYD2 | plasma membrane | extracellular |
| 17 | TNFAIP6 | extracellular | plasma membrane |
| 18 | GJB5 | plasma membrane | extracellular |
| 19 | VRK2 | mitochondrion | nucleus |
| 20 | MCM10 | nucleus | cytosol |
| 21 | IGFBP2 | extracellular | plasma membrane |
| 22 | TYMS | mitochondrion | nucleus |
| 23 | MELK | plasma membrane | nucleus |
| 24 | NDC80 | cytoskeleton | nucleus |
| 25 | OIP5 | nucleus | cytosol |
| 26 | TMEM132A | extracellular | endoplasmic reticulum |
| 27 | DUSP10 | nucleus | cytosol |
| 28 | MMP1 | extracellular | plasma membrane |
| 29 | KIF23 | cytoskeleton | nucleus |
| 30 | ASPN | extracellular | nucleus |
| 31 | CXCL2 | extracellular | nucleus |
| 32 | PRC1 | cytoskeleton | nucleus |
| 33 | CDT1 | nucleus | cytosol |
| 34 | CDC45 | cytoskeleton | nucleus |
| 35 | MCM4 | nucleus | cytosol |
| 36 | CLEC5A | plasma membrane | cytosol |
| 37 | CELSR2 | plasma membrane | extracellular |
| 38 | CCNF | cytoskeleton | nucleus |
| 39 | DLG5 | plasma membrane | cytoskeleton |
| 40 | NEK2 | cytoskeleton | nucleus |
| 41 | RCN2 | endoplasmic reticulum | nucleus |
| 42 | CHST3 | golgi apparatus | cytosol |
| 43 | ESM1 | extracellular | plasma membrane |
| 44 | ESPL1 | cytoskeleton | nucleus |
| 45 | SOX4 | mitochondrion | nucleus |
| 46 | CHEK1 | cytoskeleton | nucleus |
| 47 | C8orf4 | nucleus | plasma membrane |
| 48 | KIF18B | cytoskeleton | nucleus |
| 49 | CST1 | extracellular | nucleus |
| 50 | ATP2C1 | golgi apparatus | plasma membrane |
| 51 | AHCY | cytosol | extracellular |
| 52 | ARNTL2 | nucleus | cytosol |
| 53 | HDGFRP3 | nucleus | cytoso |
| **54** | **HOXA10** | **nucleus** | **extracellular** |
| 55 | ROBO1 | plasma membrane | extracellular |
| 56 | INPP1 | cytosol | cytoskeleton |
| 57 | CENPN | nucleus | cytosol |
| 58 | DHX34 | nucleus | cytoskeleton |
| 59 | PLK4 | plasma membrane | cytoskeleton |
| 60 | KREMEN2 | plasma membrane | endosome |
| 61 | TMEM51 | nucleus | cytosol |
| 62 | STX1A | plasma membrane | cytosol |
| 63 | UBE2C | nucleus | cytosol |
| 64 | ULBP2 | plasma membrane | extracellular |
| 65 | TOP2A | nucleus | cytosol |
| 66 | FANCI | nucleus | cytosol |
| 67 | TNFRSF10B | plasma membrane | mitochondrion |
| 68 | ST6GALNAC2 | golgi apparatus | extracellular |
| 69 | GALNT11 | golgi apparatus | nucleus |
| 70 | CHAF1B | nucleus | cytosol |
| 71 | GPR125 | plasma membrane | extracellular |
| 72 | FZD6 | plasma membrane | endoplasmic reticulum |
| 73 | NOV | plasma membrane | cytosol |
| **74** | **LHX2** | **nucleus** | **extracellular** |
| 75 | FJX1 | extracellular | golgi apparatus |
| 76 | FGD6 | golgi apparatus | cytoskeleton |
| 77 | ICAM5 | plasma membrane | extracellular |
| 78 | FAM64A | nucleus | cytosol |
| 79 | CENPF | cytoskeleton | nucleus |
| 80 | ARNT2 | nucleus | mitochondrion |
| 81 | HSPB1 | extracellular | cytoskeleton |
| 82 | IGF2BP3 | nucleus | cytosol |
| 83 | GINS1 | nucleus | cytosol |
| 84 | BIRC5 | cytoskeleton | nucleus |
| 85 | RACGAP1 | plasma membrane | cytoskeleton |
| 86 | PSMC3IP | nucleus | cytosol |
| 87 | FGFR3 | plasma membrane | extracellular |
| 88 | CDC6 | nucleus | cytosol |
| 89 | FN1 | extracellular | endoplasmic reticulum |
| 90 | SGK1 | plasma membrane | mitochondrion |
| 91 | WNT5A | plasma membrane | extracellular |
| 92 | PTGS2 | nucleus | endoplasmic reticulum |
| 93 | STK3 | cytosol | nucleus |
| 94 | CCND1 | nucleus | cytosol |
| 95 | PRKDC | nucleus | cytosol |
| 96 | DNA2 | mitochondrion | nucleus |
| 97 | ZWINT | nucleus | cytosol |
| 98 | DOCK4 | cytosol | plasma membrane |
| 99 | PTTG1 | nucleus | cytosol |
| 100 | ECT2 | plasma membrane | cytoskeleton |
| 101 | VASH2 | extracellular | cytoskeleton |
| **102** | **GADD45A** | **nucleus** | **extracellular** |
| 103 | POSTN | extracellular | golgi apparatus |
| 104 | SRD5A1 | endoplasmic reticulum | extracellular |
| 105 | CEP55 | cytoskeleton | plasma membrane |
| 106 | RBBP8 | nucleus | cytosol |
| 107 | STAR | mitochondrion | cytosol |
| 108 | RAD51AP1 | nucleus | plasma membrane |
| 109 | ABCC1 | plasma membrane | extracellular |
| 110 | PLAU | plasma membrane | extracellular |
| **111** | **HMGB3** | **nucleus** | **extracellular** |
| 112 | PKP1 | plasma membrane | nucleus |
| 113 | GJA1 | plasma membrane | mitochondrion |
| 114 | RAI14 | nucleus | cytosol |
| 115 | DSG2 | plasma membrane | extracellular |
| 116 | IRF6 | nucleus | cytosol |
| 117 | RAPGEFL1 | nucleus | cytosol |
| 118 | ZWILCH | cytosol | nucleus |
| 119 | HSPA4L | cytosol | nucleus |
| 120 | CKS1B | nucleus | cytoskeleton |
| 121 | TUFT1 | extracellular | cytoskeleton |
| 122 | ITGAV | plasma membrane | extracellular |
| 123 | B4GALT6 | golgi apparatus | plasma membrane |
| 124 | KIF2C | cytoskeleton | cytosol |
| 125 | NOX4 | plasma membrane | nucleus |
| 126 | TFRC | plasma membrane | extracellular |
| 127 | PBK | nucleus | cytosol |
| 128 | TK1 | cytosol | mitochondrion |
| 129 | PLA2G3 | plasma membrane | extracellular |
| 130 | ASPM | nucleus | plasma membrane |
| 131 | FAP | plasma membrane | extracellular |
| 132 | LAMB1 | extracellular | endoplasmic reticulum |
| 133 | PPIF | mitochondrion | cytosol |
| 134 | STAP2 | plasma membrane | cytosol |
| 135 | BUB1B | cytoskeleton | nucleus |
| 136 | HJURP | nucleus | mitochondrion |
| 137 | DTL | cytoskeleton | nucleus |
| 138 | MEST | extracellular | endoplasmic reticulum |
| 139 | FERMT1 | plasma membrane | cytoskeleton |
| 140 | KIF20A | cytoskeleton | nucleus |
| 141 | HOXC6 | nucleus | cytosol |
| 142 | NPL | cytosol | plasma membrane |
| 143 | TTK | nucleus | cytoskeleton |
| 144 | NCAPG | nucleus | cytosol |
| 145 | NUSAP1 | nucleus | cytoskeleton |
| 146 | KRT32 | cytosol | extracellular |
|  |  |  |  |
|  |  |  |  |
