# Supplementary material for: Nuclear exosome HMGB3 secreted by nasopharyngeal carcinoma cells promotes tumour metastasis by inducing angiogenesis
Source: Cell Death Dis. 2021 May 28;12(6):554. doi: 10.1038/s41419-021-03845-y (PMC8163785; doi:10.1038/s41419-021-03845-y)
Supplement: Supplementary file 2 — Relationship between HMGB3 expression and clinicopathological characteristics of NPC patients [file 41419_2021_3845_MOESM2_ESM.docx]

**Table S2. Relationship between HMGB3 expression and clinicopathological characteristics of NPC patients**

| **Clinicopathological**  **characteristics** | **n** | **Low**  **expression** | **High**  **expression** | χ***2*** | ***P-*value** |
| --- | --- | --- | --- | --- | --- |
| **Total** | 129 |  |  |  |  |
| **­Gender** |  |  |  | 0.163 | 0.687 |
| Male | 99 | 73 | 26 |  |  |
| Female | 30 | 21 | 9 |  |  |
| **Age (years)** |  |  |  | 1.501 | 0.220 |
| ≤47 | 66 | 45 | 21 |  |  |
| ＞47 | 63 | 49 | 14 |  |  |
| **Tumor diameter(cm)** |  |  |  | 2.597 | 0.107 |
| **≤**1 | 104 | 79 | 25 |  |  |
| **＞**1 | 25 | 15 | 10 |  |  |
| **Lymph node metastasis** |  |  |  | 0.115 | 0.735 |
| Absent | 36 | 27 | 9 |  |  |
| Present | 93 | 67 | 26 |  |  |
| **Recurrence** |  |  |  | 7.119 | **0.008**** |
| Absent | 69 | 57 | 12 |  |  |
| Present | 60 | 37 | 23 |  |  |
| **TNM** |  |  |  | 5.673 | **0.017*** |
| Ⅰ~Ⅱ | 70 | 57 | 13 |  |  |
| Ⅲ~Ⅳ | 59 | 37 | 22 |  |  |

*Statistical analyses were performed by the Pearson χ2 test. P<0.05 was considered significant.
