# Supplementary figures and images for: Nuclear exosome HMGB3 secreted by nasopharyngeal carcinoma cells promotes tumour metastasis by inducing angiogenesis
Source: Cell Death Dis. 2021 May 28;12(6):554. doi: 10.1038/s41419-021-03845-y (PMC8163785; doi:10.1038/s41419-021-03845-y)

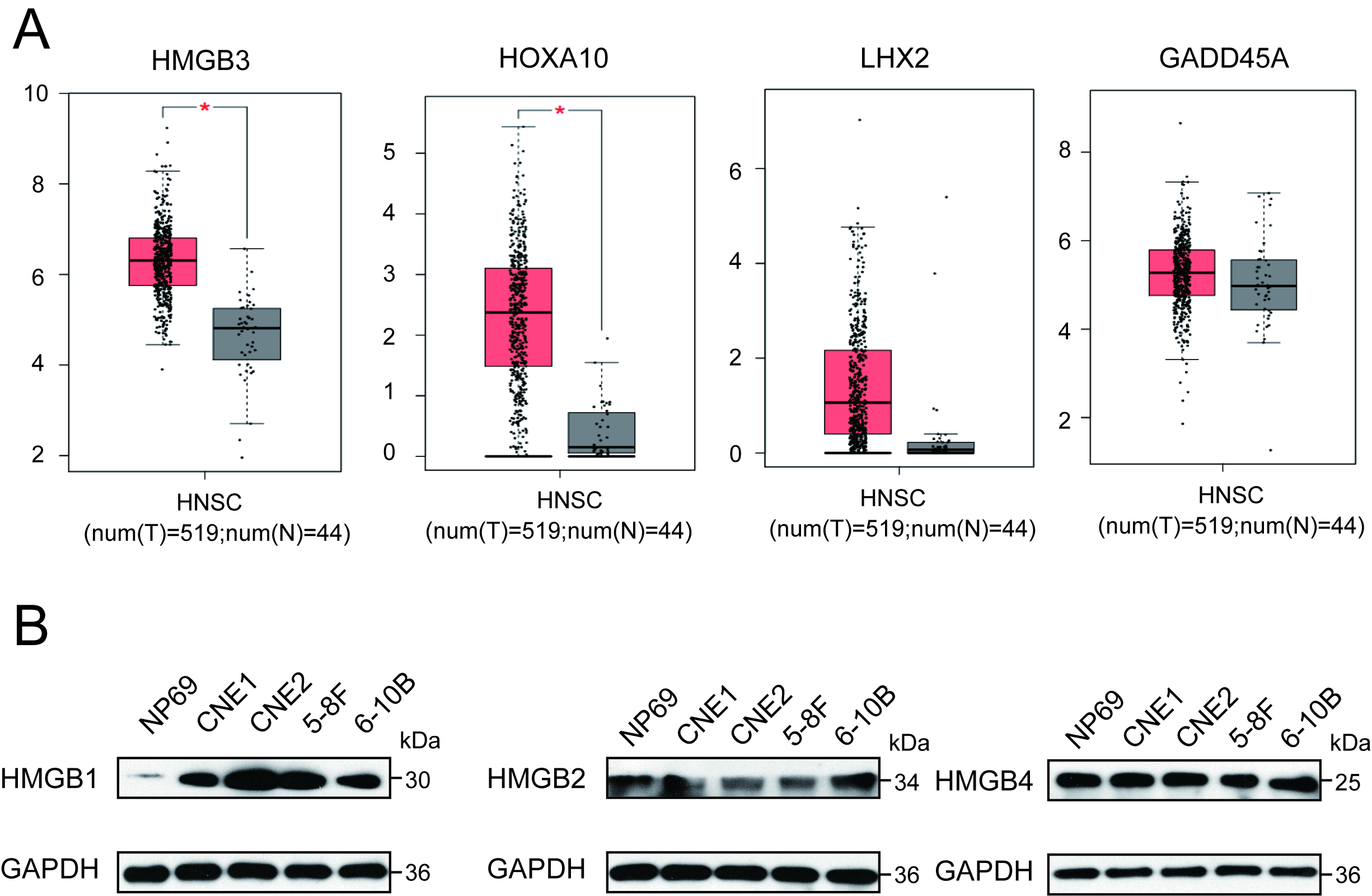

Supplement: Supplementary file 3 — The expression of the four genes in the TCGA database and the expression in cell lines of the HMGB family. [file 41419_2021_3845_MOESM3_ESM.tif]

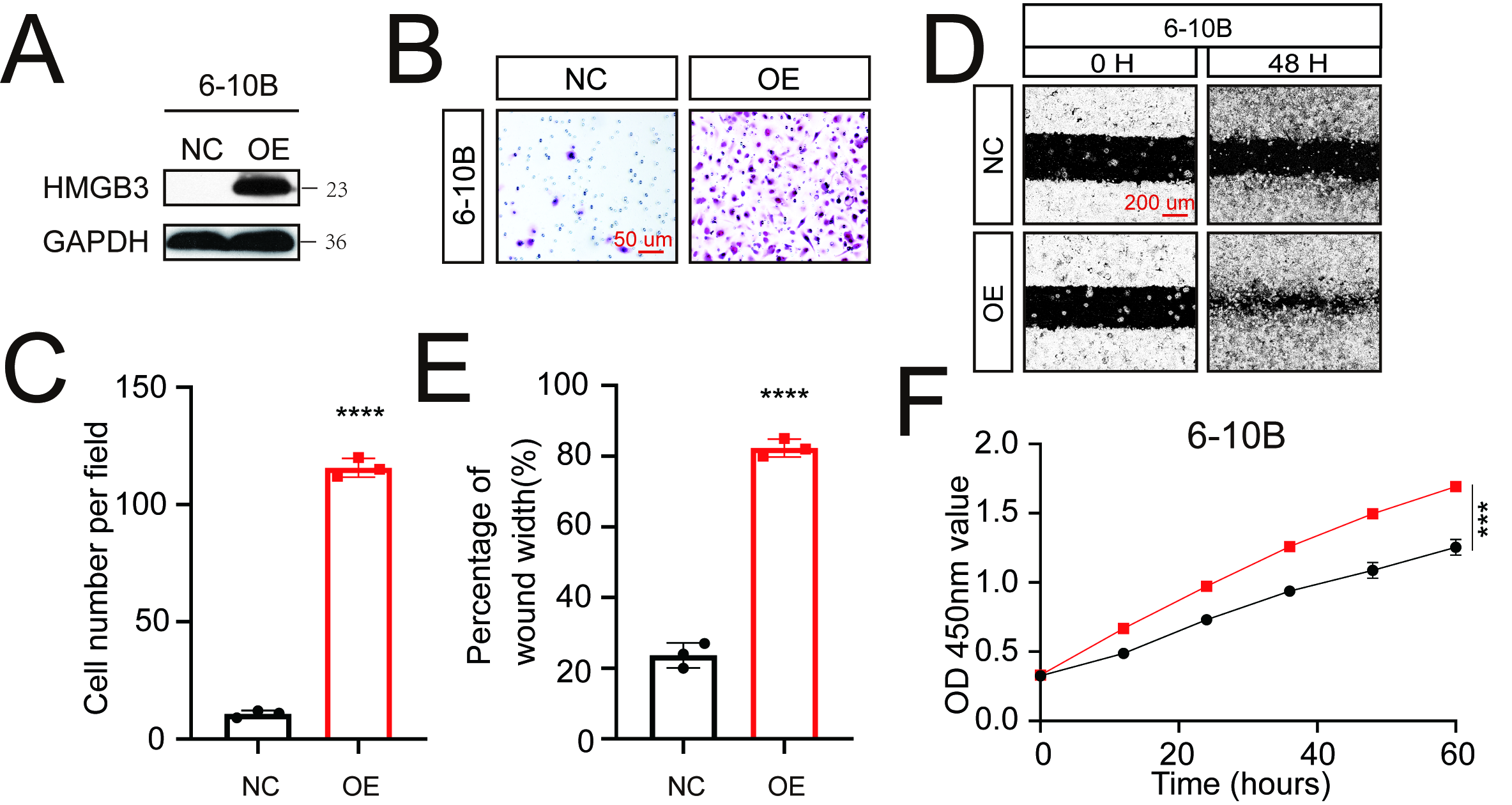

Supplement: Supplementary file 4 — HMGB3 overexpression promote NPC cell proliferation and migration in vitro. [file 41419_2021_3845_MOESM4_ESM.tif]
